# Role of structural specificity of ZnO particles in preserving functionality of proteins in their corona

**DOI:** 10.1038/s41598-021-95540-3

**Published:** 2021-08-05

**Authors:** Urvashi Singh, Zeeshan Saifi, Mridul Kumar, Armin Reimers, Soami Daya Krishnananda, Rainer Adelung, Martina Baum

**Affiliations:** 1 Microwave Physics Lab, Department of Physics and Computer Science, Dayalbagh Educational Institute (Deemed to be University), Dayalbagh, Agra India; 2 Functional Nanomaterial Group, Institute for Material Science, Kiel University, Kiel, Germany

**Keywords:** Physics, Biological physics, Nanoscience and technology, Techniques and instrumentation, Characterization and analytical techniques

## Abstract

Reconfiguration of protein conformation in a micro and nano particle (MNP) protein corona due to interaction is an often-overlooked aspect in drug design and nano-medicine. Mostly, MNP-Protein corona studies focus on the toxicity of nano particles (NPs) in a biological environment to analyze biocompatibility. However, preserving functional specificity of proteins in an NP corona becomes critical for effective translation of nano-medicine. This paper investigates the non-classical interaction between insulin and ZnO MNPs using a classical electrical characterization technique at GHz frequency with an objective to understand the effect of the micro particle (MP) and nanoparticle (NP) morphology on the electrical characteristics of the MNP-Protein corona and therefore the conformation and functional specificity of protein. The MNP-Protein corona was subjected to thermal and enzymatic (papain) perturbation to study the denaturation of the protein. Experimental results demonstrate that the morphology of ZnO particles plays an important role in preserving the electrical characteristics of insulin.

## Introduction

The size specificity of nanoparticles (NPs) is often considered a primary factor controlling protein adsorption^1–3^. Additionally, protein adsorption on NPs can also be influenced by their surface morphology, which is characterized by large surface area and curvature^1–4^. This manuscript primarily aims at examining the roles of the structural specificity and physicochemical properties of micro and nano particle (MNP)—protein corona in Nano-Bio interaction.

Interactions of protein with other molecules disrupt the electrostatic interaction among the residues which further leads to conformational changes and in turn affects the protein functions^5, 6^. The electrostatic interactions can also vary by diverse processes such as binding of charged ligands, substitutions of amino acids during site-directed mutagenesis and also by changes in the tertiary or quaternary structural configurations of the protein and other bio-molecules^7^. Therefore, electrostatic interaction plays a crucial role in stabilizing protein^7, 8^. This motivates to use electrical parameters of proteins for assessing their structural conformation.

Among the several physico-chemical properties^9^ which affect the behavior of molecules, the dielectric constant is an important one. Study of the dielectric properties of proteins is of great interest in the microwave region due to its spatial variation in orientational polarization leading to temporal change in the dielectric relaxation^10–12^. The dielectric constant of proteins depends on the distribution of charge residues, side chains and their packing. Tightly and loosely packed regions show low and high dielectric constants respectively^13, 14^. Therefore the physical state of the bio-macromolecules can be characterized using the dielectric studies which could help to understand the stability and interaction of protein in a biological media^13, 14^.

In-depth protein dynamics like slow and fast spatiotemporal local or collective molecular motions produced by charge residues can give new understanding about the Nano-Bio interface^15^. To understand the phenomenon taking place at the molecular level, a theoretical model was developed where it is assumed that the MNP protein complex suspended in a buffer medium is governed by binding, damping and driving forces, which arise due to chemical bonding, buffer drag and applied electric field respectively (See Materials and Methods section). By solving the governing differential equations, a theoretical expression for the mechanical vibration frequency was derived, which is a function of the dielectric constant and the dipole moment (See Materials and Methods section). Using microwave resonant technique (See Methods and Supplementary Section), we have studied the protein along with MNPs under various perturbing conditions like adding a denaturing agent and applying thermal stress. The results are further validated by conventional techniques like Dynamic Light Scattering (DLS) and UV–Vis spectroscopy and Differential Scanning calorimetry (DSC).

We report here the results of two studies. First, the interaction of insulin and ZnO, the choice of the system is based on the demonstrated compatibility of ZnO with insulin^4^. Second, to understand the conformational changes using protein cleaving enzyme papain. Papain is a heat-resistant enzyme that cleaves peptide bonds of amino acids including leucine, glycine and cysteine^16, 17^. Since, insulin contains a good amount of these amino groups, papain severely cleaves it. In our study, the role of papain in denaturing insulin has been observed by measuring the change in dielectric properties and zeta potential. In addition, we have also studied the thermal variation of electrical properties of protein complex. Results from these studies demonstrate the effect of morphologically different MNPs on preserving the electrical configuration of proteins.

## Results

### Dielectric constant of insulin and papain

**Figure 1:**
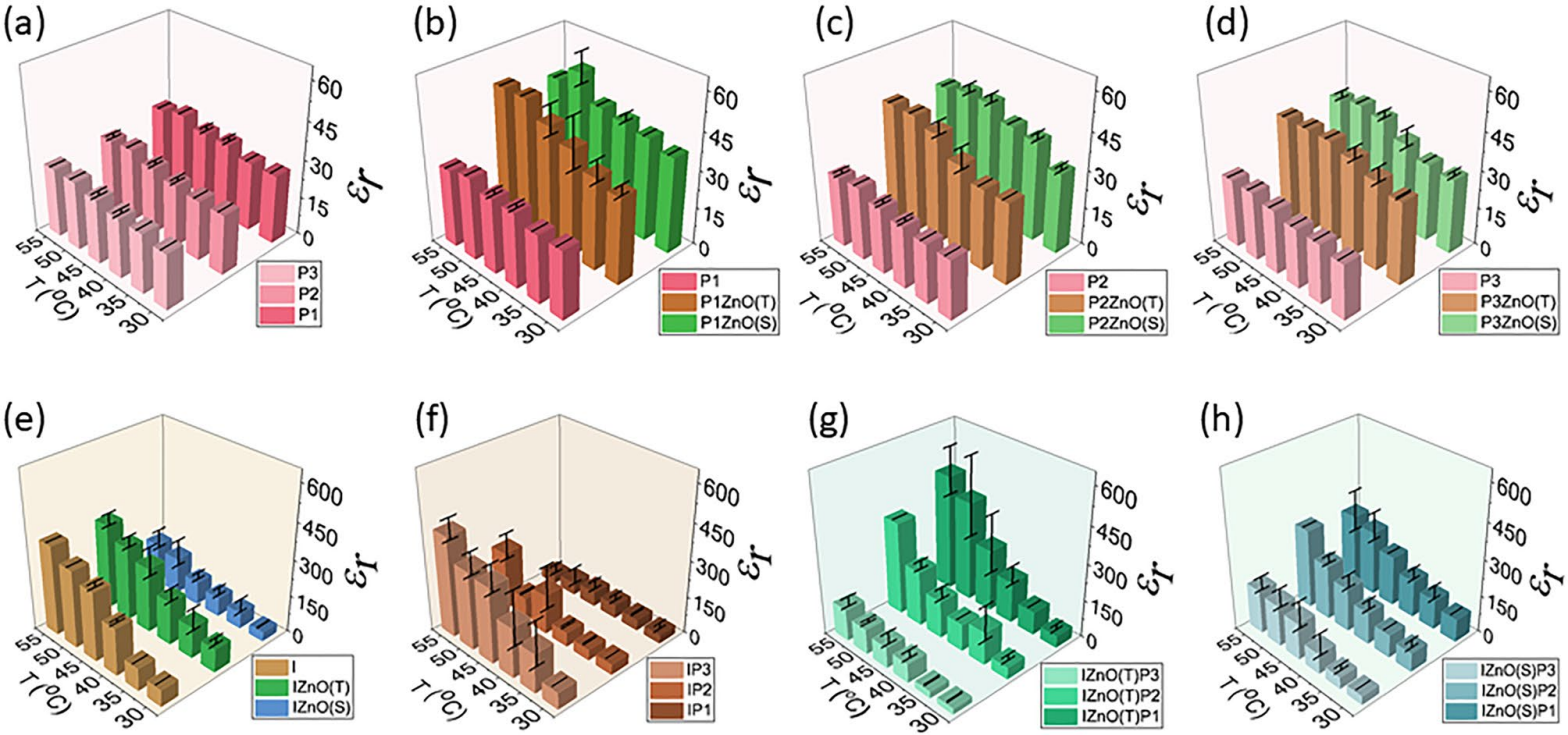
Dielectric constant of (**a**) Papain of three dilutions (**b**–**d**) and mixed with ZnO(S) and ZnO(T) (**e**) Insulin and Insulin mixed with ZnO(S) and ZnO(T) (**f**) Insulin mixed with three different dilutions of papain (**g**) Insulin mixed with ZnO(T) and Papain (**h**) Insulin mixed with ZnO(S) and Papain. Here the abbreviations P1, P2 and P3: Higher, Intermediate and lower concentration of Papain and I: Insulin and εr: dielectric constant.

Thermal variations directly affect the viscosity, intra-molecular interaction, conformational state variations, and the stability of dimers in proteins^18–20^. We analyzed the thermal effects on the dielectric constant of insulin and papain in the temperature range between 30 and 55 °C at intervals of 5 °C. For insulin, the dielectric constant was 68 at 30 °C and an increase in temperature was directly proportional to an increase in dielectric constant, with a maximum of 370 at 55 °C (Fig. 1). For papain (high concentration i.e., 10 mg/ml) at 30 °C, the dielectric constant was 27 which increased to 31 on heating to 55 °C, and the values for intermediate temperature are shown in Fig. 1. Diluted samples of papain (see methods) showed that the dielectric constant was directly proportional to the papain concentration, as the dielectric constants were observed to be 24 (30 °C) and 22 (30 °C) for the dilution factors ½ and ¼ respectively. However, on heating the diluted samples of papain a similar increasing trend in dielectric values was observed, the results of which are shown in Fig. 1. The trend of increasing dielectric constant with temperature agrees with literature reported on polymers (including papain)^21^. It is evident from the observations and subsequent repeat experiment that insulin is more sensitive to thermal variation as compared to papain, as reflected in the dielectric constant variations. In the present work, we have performed two runs to ensure the repeatability of the dielectric measurements. The average of which has been plotted in Fig. 1. It can be seen from the figure that the pure samples show the same values on both runs at the initial temperature. However, the dynamic nature of the insulin at intermediate temperature adds to the variation in dielectric values. This can be related to the monomer–dimer equilibrium in insulin which is sustained at room temperature and dissociates on high temperature (> 45 °C)^18–20^.

### ZnO with insulin and papain

To test the physicochemical preserving nature of ZnO on insulin and papain, and the effect of particle morphology on this preserving property, two morphologically different ZnO particles, Tetrapodal micro particles (ZnO(T) of size ~ 15 µm)^22^ and ii) Spherical nanoparticles (ZnO(S) of size ~ 100 nm) were used. Besides surface morphology, ZnO(T) is highly crystalline in nature as compared to ZnO(S). Here we analyzed the effect of ZnO particles on the dielectric constant of insulin and papain, at different temperatures. When ZnO(S) particles were mixed with insulin, the dielectric constant of the complex (Insulin + ZnO(S)) was 40 ± 2 (30 °C) and upon heating, the values increased to 167 ± 38 (55 °C). In this case, the rate of increase was not as significant as it was for pure insulin (i.e., 68 at 30 °C and 369 at 55 °C). When ZnO(T) was mixed in insulin, the dielectric constant was found to be 95 ± 14 (30 °C) which, upon heating increased to351 ± 25 (55 °C). Interestingly, the complex of Insulin + ZnO(T) exhibited similar dielectric behaviour as that of pure insulin. All the dielectric constant values for the Fig. 1 are given in the Supplementary file 3.

On mixing ZnO particles in papain, it was observed that the dielectric constant of the complex (Papain + ZnO) increased for all dilutions of papain samples (see Fig. 1). This increment was more for samples with ZnO(S) as compared to samples with ZnO(T). Like all other samples, in this case also, the increase in dielectric constant was directly proportional to the increase in temperature. As discussed above papain being thermally resistive shows less increment in its dielectric constant on heating (as compared to insulin), the complex of ZnO and papain also showed a similar pattern (see Fig. 1). Above results show that the two proteins insulin and papain having different properties (See Table 5 of Supplementary File 1) and thus demonstrating different interaction with MNPs.

### Mixing papain and insulin

Papain denatures other proteins and therefore falls under the category of protease^16^. In our earlier work, we reported the denaturing effect of papain on egg proteins, plant protein, and insulin. We found that the dielectric constant decreases on adding papain to proteins^23^. Here, we have extended our investigation to understand the effect of temperature variation in samples of insulin mixed with different concentrations of papain. It was observed that on mixing papain with insulin, the net dielectric constant of the complex decreased significantly. This reduction was proportional to the amount of papain added. Further, on increasing the temperature for samples mixed with a higher concentration of papain, the dielectric constant linearly increased and reached a maximum value of 40 at (45 °C) and then eventually decreased on further heating, this effect was confirmed by performing a control measurement. In the second experiment the maxima was observed at 50 °C and then the dielectric values decreased. This variation was absent in samples mixed with a lower concentration of papain. In fact, for a lower concentration of papain, the dielectric variations on heating were not significantly different from pure insulin.

### Mixing ZnO, with insulin and papain

After performing the baseline analysis for assessing the effect of temperature and presence of ZnO and papain on insulin, we further extended the analysis to study the thermal variations of the denatured complex of insulin and papain and to study the effect of ZnO particles on the same. Having observed that the addition of papain and ZnO decreases and increases the dielectric values of insulin respectively, we investigated the combined effects of papain and ZnO on insulin. In the mixture containing insulin, papain and ZnO(T), papain had a denaturing effect on insulin, ZnO(T) exhibited a monotonic increase in the dielectric values of the denatured insulin with temperature. The dielectric constant for high temperatures was higher compared to the samples of papain + insulin without ZnO, demonstrating the effect of ZnO(T) on the thermal behavior of the protein complex. Doing the same analysis on samples of insulin + papain mixed with ZnO(S), a monotonic increase of the dielectric values (94 to 291 ± 85 for higher, 77 ± 16 to 325 for intermediate and 36 to 178 ± 27 for lower concentrations, in the temperature range from 30 °C to 55 °C, respectively) was observed. This change is significantly lesser in comparison to ZnO(T) mixed with insulin + papain (49 ± 12 to 475 ± 97, 49 ± 12.1 to 377 and 25 ± 3 to 130 ± 20 for higher, intermediate and lower concentration respectively in the temperature range 30 °C to 55 °C). We can observe from the figure that the pure samples show almost negligible variation on performing repeat dielectric measurements as compared to mixed samples. It is also to be reported that, when papain was mixed with MNPs, the dielectric variation at high temperature was less as compared to the samples in which papain was mixed with insulin, suggesting the proteolytic action of papain on insulin^16, 17^.

### Thermal effects on mechanical vibration of proteins as pure solutions

The calculated frequencies of mechanical vibration through the theoretical model based on dielectric constant, and dipole moment (see Methods) indicate that the order of mechanical vibrations was 10^3^ times less than the applied field (of the resonant antennae used as a probe). Since the resonant frequency of antenna was ~ 6.4 × 10^9^ Hz, the calculated mechanical vibrations of protein samples were in the MHz range, which corresponds to time interval in the ~ µs range. The order of time matches well with the findings of Ugo Mayor et al., in which the group reported that the collapse of protein into an intermediate native α-helical secondary structure (a major constituent of denatured state) happens in time scale of microseconds^24^.

**Figure 2:**
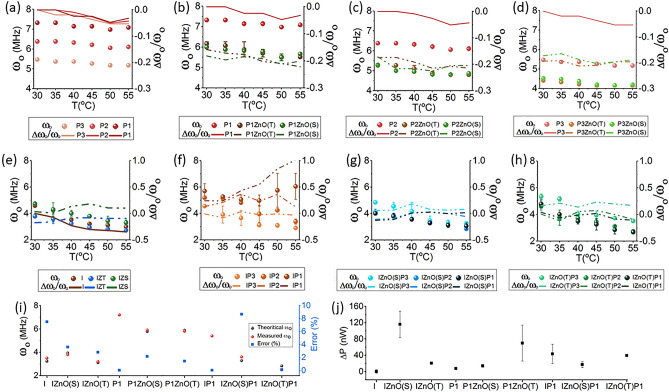
Theoretically proposed Mechanical Frequency (ω_o_) of (**a**) Papain of three dilutions (**b**–**d**) and mixed with ZnO(S) and ZnO(T) (**e**) Insulin and Insulin mixed with ZnO(S) and ZnO(T) (**f**) Insulin mixed with three different dilutions of papain (**g**) Insulin mixed with ZnO(S) and Papain. (**h**) Insulin mixed with ZnO(T) and Papain (Here the average of theoretical mechanical frequency is plotted. Where the bar shows two runs executed as in case of dielectric constant) (**i**) Measured and Theoretical mechanical frequency using Spectroscopic technique and theoretical proposed model respectively and the corresponding relative error with the theoretical (Here the value corresponding to 45 °C and the case at 50 °C discussed in Supplementary file 2) (**j**) the Average power changed of sample, the Experiment was performed twice. So, the bars indicate the variation of two runs. Here P1, P2 and P3: Higher, Intermediate and lower concentration of Papain and I: Insulin.

On increasing the temperature, the mechanical frequency of insulin decreased, this can be viewed as an effect resulting from an increased surface area when a protein unfolds. Since the frequency of vibrations (from the theory of oscillators) depends on mass and length, F.S. Legge et al. performed molecular dynamics (MD) simulations to see the effect of temperature on the unfolding of insulin, by analyzing the unfolding through increase in distance between two residues (residue 5 and residue 13) of insulin^25^. Papain being thermally resistive showed very nominal variation in mechanical frequency on heating. However, we observed that the mechanical frequencies were significantly different for different concentrations (Fig. 2).

### Thermal effects on mechanical vibration of proteins in presence of ZnO

The mechanical frequency of papain + ZnO(T) and papain + ZnO(S) was less as compared to the relevant same concentration of the pure solution of papain. The thermal inactivity of papain was observed, as the mechanical frequency did not change much on increasing the temperature as shown in Fig. 2. In contrast, for samples of insulin + ZnO(S), the mechanical frequencies (for all temperatures) increased as compared to pure insulin, whereas, for insulin + ZnO(T) the observed mechanical frequencies were lesser than the frequencies observed for pure insulin. Other than pure samples and samples mixed with ZnO, the mechanical frequencies were also computed for the combination of all complexes as it was done for the case of dielectric constant. Significantly, when insulin was mixed with the higher concentration of papain, the mechanical frequency was nearly constant till 45 °C and then, a sharp increase was observed on further heating to 50 °C. This instant increase in the mechanical frequency indicates a loss in the mass of protein or fragmentation of protein complex (reduced length). However, in the presence of ZnO, increment in temperature caused a monotonic reduction in the mechanical frequency with no anomalies observed at higher temperatures, suggesting the increase in the mass/length of the MNPs-protein complex.

### Verification of theoretically calculated mechanical frequencies

To verify the theoretically predicted values of mechanical frequencies which were observed in the MHz range, frequencies of all samples in the close neighbourhood of theoretical values were pumped into the samples using vector signal generator, with the pumped power kept fixed at -50 dBm which corresponds to 10 µWatts. If the input frequencies match with the natural frequency of the sample, power is absorbed due to resonant interaction. Using this fact, we found that for insulin the frequency was 3.5 MHz (45 °C) which is approximately close to the above-predicted value of 3.237 ± 0.304 MHz. Similarly, for other samples also, the observed frequencies were close to the calculated values with a deviation of no more than 1 MHz in any case. On performing the second run, we found that the frequencies at which power absorption was noticed remained the same, though, the magnitude of absorption was different. Mean power absorbed and the standard deviation for all samples are shown in Fig. 2.

### Validation of results using conventional UV–Vis spectroscopy

UV–Vis absorption spectroscopy is widely used to analyze the interaction between proteins and nanoparticles and also to study the conformational changes (like the formation of the nanoparticles-protein corona)^26, 27^. Events like the unfolding of proteins, mutual interaction between a diverse variety of proteins, and their binding with nanoparticles can be interpreted based on careful analysis of the absorbance curve^27^. The wavelength corresponding to the peak absorbance at varying temperature (20 °C, 30 °C, 40 °C and 50 °C) was measured for all samples, (Table 1). Peak absorbance wavelength for insulin and papain, as well as for ZnO(T) and ZnO(S) were all found to be close to 282 nm which did not change much on heating the samples^28–31^. A perceivable redshift (i.e. peak absorbance shift towards higher wavelength) of 5.0 nm and 3.0 nm was observed on mixing ZnO(S) and ZnO(T) in insulin respectively. Whereas for papain + ZnO this shift was reduced to 2.0 nm and 1.0 nm for ZnO(S) and ZnO(T) respectively. A redshift suggests enhanced adsorption of proteins on the surface of the particle^31^. Absorbance peak at 379 nm and 377 nm were found for ZnO(T) and ZnO(S) respectively but no significant change with temperature was observed.

**Table 1:** The measured wavelength corresponding to the absorbance peak of all samples at various temperatures (20 °C, 30 °C, 40 °C and 50 °C) using UV spectrophotometer.

	Wavelength (nm)										
	Pure samples				Mixed samples						
T (°C)	ZnO(S)	ZnO(T)	I	P	IZnO(S)	IZnO(T)	PZnO(S)	PZnO(T)	IP	IZnO(S)P	IZnO(T)P
20	282*	283	285	283	287	286	284	284	285	284	285
30	282	282	284	282	287	284	282	282	284	282	284
40	282	282	284	282	286	287	283	282	284	282	285
50	282	282	284	282	285	285	283	282	284	282	285

### Measurement of electrokinetic potential and analyzing surface charge

The conformational changes in protein complexes are known to further affect the surface charge properties like the electrokinetic potential of the slipping plane^32^. Zeta potential is the key parameter to scale the electrostatic interaction in a colloidal dispersion and is a measure of the electrical stability of the colloid^32^. For pure insulin the Zeta potential was − 12.3 mV which was close to − 15 mV as reported^33^, whereas, for papain the Zeta potential was only 6.09 mV (See Table 2). A positive zeta potential generally evinces the presence of more positive charges in contrast to the negative charges. Papain enzyme is composed of 24 positively charge amino groups, outnumbering negatively charged amino groups which are 15, and the zeta potential values reflect the same^34^. On studying the Zeta potential of insulin + ZnO we found an increase in Zeta potential. For the complex of insulin and tetrapodal particles, the Zeta potential was − 16.7 mV and for the complex of insulin and spherical particles, Zeta potential was − 18.13 mV. These observations correlate with the dielectric variations studied earlier in this manuscript, where we found that the relative change (with respect to pure insulin) in the dielectric constant of insulin + ZnO(T) was small as compared to insulin + ZnO(S). Zeta potential values of papain + ZnO also show that spherical ZnO causes a substantial change in the Zeta value (15.2). However, the Zeta Potential of papain + ZnO(T) showed only a slight increase to 6.68 mV from 6.09 mV (pure papain).

**Table 2:** Measured Zeta potential (DLS data) of the samples.

	Zeta potential (mV)										
	Pure samples				Mixed samples						
	ZnO(S)	ZnO(T)	I	P	IZnO(S)	IZnO(T)	PZnO(S)	PZnO(T)	IP	IZnO(S)P	IZnO(T)P
Run1	− 10.9	− 19.9	− 12.3	5.98	− 17.3	− 16.2	15.2	6.38	13.5	13.3	9.96
Run2	− 10.4	− 21.2	− 11.7	6.68	− 18.3	− 16.7	15.2	6.84	12.9	13	11
Run3	− 11.4	− 16.4	− 12.9	5.62	− 18.8	− 17.4	15.3	6.84	13.4	12.4	11.7
Avg	− 10.9	− 19.1	− 12.3	6.09	− 18.1	− 16.7	15.2	6.68	13.2	12.9	10.88

### Study of thermodynamic parameters of proteins under thermal transition using DSC

Results were further validated using an analytical technique through thermodynamic investigation that directly calculates the change in enthalpy (ΔH) and specific heat (ΔC_p_) of a thermal transition. This change in enthalpy correlates to the denaturation in terms of heat required for unfolding of a protein^35^. For endothermic process, ΔH is a positive value and for exothermic, ΔH is negative. Denaturation involves the uptake of heat required in endothermic reaction^35^. The positive endothermic DSC peak was found in case of protein and their mixtures with MNPs. Further, thermograph analysis was performed based on the transition peaks. Also, ΔH and the temperature corresponding to peak value in the thermograph (T_m_) for heat denaturation were 69.67 ± 10.63 (J/g °C), (52.78 ± 1.49) °C for insulin and 103.28 ± 22.73 (J/g °C), and (59.43 ± 4.16) °C for papain respectively. ΔH value for insulin mixed with MNPs was closer to the insulin as compared to the insulin mixed with papain (See Table 3). Maximum enthalpy change is found for Insulin + papain which validate the volatile behavior of papain as observed in dielectric studies.

**Table 3:** DSC measurements of samples.

S. No	Sample	(ΔH ± δ) J/g °C	(T_m_ ± δ) °C	(ΔCp ± δ) J/g °C	(T_g_ ± δ) °C
1	I	69.67 ± 10.63	52.78 ± 1.49	2.10 ± 0.08	37.83 ± 0.40
2	P	103.28 ± 22.73	59.43 ± 4.16	3.64 ± 0.42	41.14 ± 1.49
3	ZnO(S)	8.08 ± 1.50	49.82 ± 1.17	0.18 ± 0.02	38.23 ± 5.75
4	ZnO(T)	7.13 ± 1.07	40.82 ± 4.47	0.53 ± 0.30	33.44 ± 0.47
5	IZnO(S)	62.15 ± 1.25	46.61 ± 0.01	2.19 ± 0.05	34.60 ± 0.07
6	IZnO(T)	38.26 ± 1.22	50.46 ± 0.51	2.08 ± 0.61	37.47 ± 1.06
7	PZnO(S)	107.70 + 7.01	51.26 ± 0.33	4.24 ± 0.03	37.83 ± 0.27
8	PZnO(T)	125.35 ± 11.45	53.08 ± 0.18	4.78 ± 0.08	39.19 ± 0.09
9	IP	213.35 ± 64.19	58.58 ± 0.02	6.38 ± 1.79	43.42 ± 0.86
10	IZnO(S)P	83.67 ± 10.94	56.13 ± 5.18	3.10 ± 0.11	40.69 ± 2.08
11	IZnO(T)P	120.44 ± 16.36	53.10 ± 0.16	4.22 ± 0.55	38.64 ± 0.21

ΔH, average change in enthalpy; Tm, average temperature corresponding to peak value in DSC thermograph curve having range 30–100 °C; ΔC_p_, average change in specific heat; T_g_, average temperature at half Cp extrapolated; δ, variation of two independent runs. Scan rate 20 °C/min.

## Discussion

**Figure 3:**
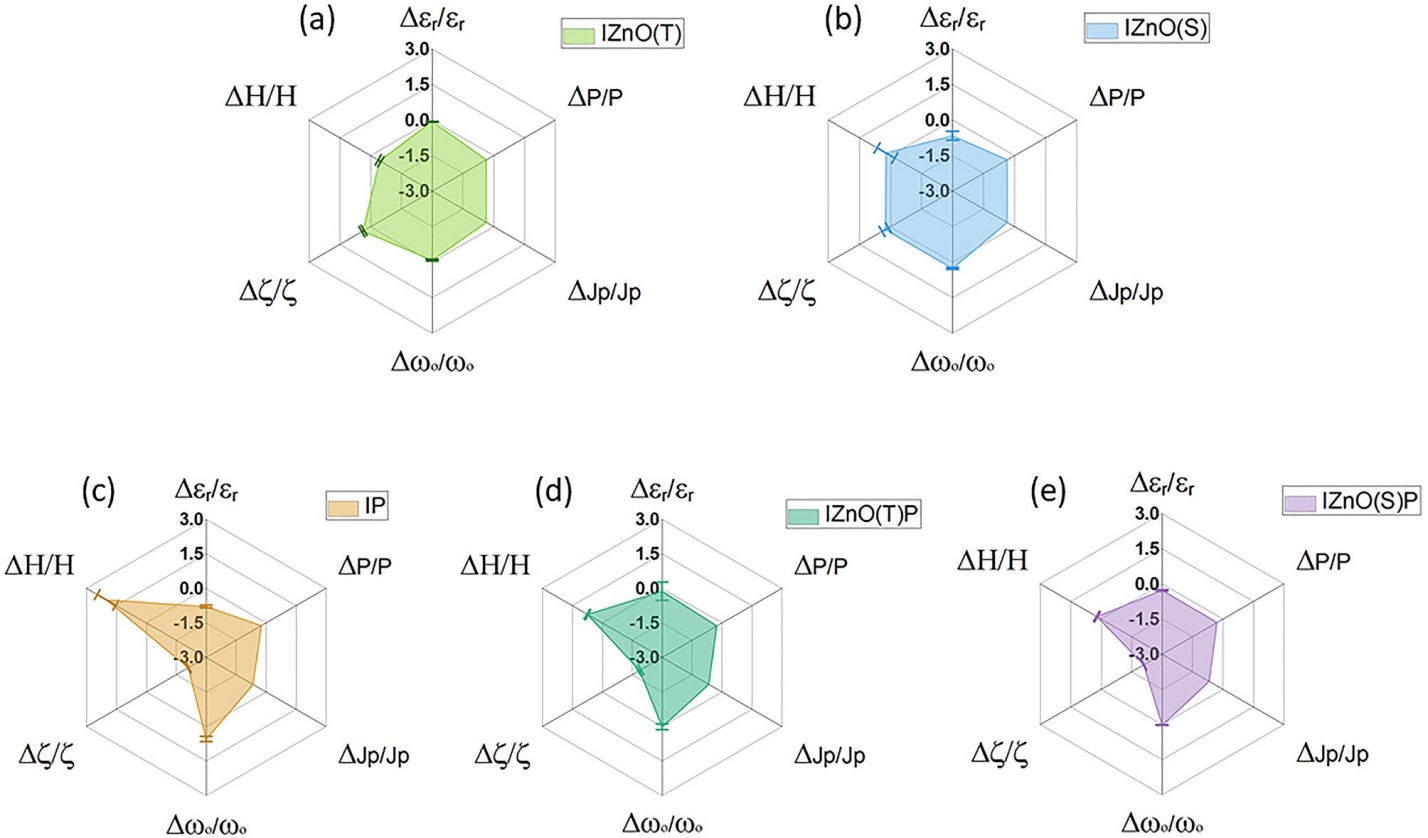
Radar chart of correlation of physical parameters (The abbreviation ε_r_, P, Jp, ω_o_, ζ and H are used for physical parameters dielectric constant, dipole moment, polarization current density, mechanical frequency zeta potential and enthalpy), These Y chart values are relative change (with respect to Insulin) for various samples which suggest the deviation from the physical parameter of Insulin. Norman cloture for papain sample P is used here because P is carried with different concentration in these techniques. Here for microwave spectroscopy P1 (Highest concentration) is used and the lower dilutions were used for zeta potential measurement because high concentrations are not suitable in these studies due to turbulence. The detail protocol of sample preparation was discussed in the Supplementary file 1. Although, mixing ratio is same in all.

The above stated results indicate variation in dielectric constant due to atomic and molecular interaction owing to temperature dependent protein unfolding or denaturation. The dipole fluctuation depends on both collective large-scale motions^15^ and local motions^15^, therefore, probing the dipole fluctuation or the dipole moment through measurement of dielectric constant can offer deeper understanding on the interaction mechanism of MNPs and proteins and their unfolding. In the type of system which we dealt with, it can be assumed that the MNP-protein complex is acted upon by binding force due to chemical bonding, driving force due to applied field and the damping force offered by the buffer in which MNP-protein complex was dispersed. The mechanical frequency of the protein complex was calculated using the proposed theoretical model based on the dielectric constant and dipole moment (See Methods Section). This parameter aided in understanding the interaction taking place at the molecular level. Figure 3 illustrates the interaction of insulin with MNPs and papain. Physical properties such as dielectric constant, dipole moment, polarization current density and zeta potential of Insulin + ZnO(T) (IZnO(T)) are closer to insulin as compared to Insulin + ZnO(S) (IZnO(S)), Insulin + ZnO(S) + Papain (IZnO(S)P) and Insulin + ZnO(T) + Papain (IZnO(T)P). This study concludes that shape and surface morphology of MNPs can affect or preserve the electrical configuration of protein. The ZnO(T) structures can preserve the polarity and spatial-surface charge distribution of protein, therefore, can be effective carriers and preservatives for insulin. Overall, this study offers novelty in understanding bio-molecular interaction, the variation in electrical properties of protein during MNPs interaction indicating sensitivity to atomic and molecular interaction changes. Thus, exploring the electrical properties of the MNP-Protein complex and their optimal variation compared with the corresponding pure protein sample can provide a better understanding of the functionality, stability, and interaction, etc. Dielectric results were further validated using DSC, the enthalpy measurements from DSC agree with the dielectric results. This has implications for nano-drug design, where nanoparticles tend to change the surface electrical properties of MNP-Protein corona and thereby change the functional properties of proteins. Such studies have the potential to overcome or address the slow or not very successful translation of nano-medicine^36–40^.

## Material and methods

### Materials

Insulin Humolog (Powder type) purchased from Sigma Aldrich was diluted in HEPES Buffer (50.7 ml of distilled water, 1.3 ml of HEPES Buffer) at a final concentration of 6.9 mg/ml. For understanding the NP-Protein interaction, ZnO(S) and ZnO(T) were used. ZnO(S) (< 100 nm) is purchased from Sigma Aldrich whereas ZnO(T) are synethised using flame transport synthesis method by Functional Nano-material group, Kiel University, Germany^41^. The NPs are prepared in distilled water at a final concentration 3.45 mg/ml. Further studying denaturation, the strong protease enzyme (Papain) purchased from BIOENZYME and prepared in distilled water and cleaned through vacuum pump. Three different dilutions (1/2^n^) of the papain P1 (10 mg/ml), P2 (5 mg/ml) and P3 (2.5 mg/ml) have been selected for dielectric measurements. All solutions were prepared freshly when used for measurements. All the sample concentration discussed above is used for dielectric measurement. However, Electrophoretic and UV–VIS spectroscopy cannot work with high concentration because of creating turbulence. Thus 100 µl of each solution (ZnO(S), ZnO(T), P1 and insulin) mixed with 900 µL of distilled water (for ZnO(S), ZnO(T) and P1) and HEPES buffer (for Insulin). For all techniques, the mixing ratio was fixed i.e., 2:1 for Insulin and NPs, 10:1 for insulin and papain, 8:1:4 for insulin, papain and NPs. Detailed protocol of sample preparation and mixing ratio is discussed in supplementary file (See Supplementary file 1).

### Methods

The dielectric measurements performed with the coaxial fork type probe designed at 6.45 GHz. The near field region of probe is less than 10 cm thus does not sense the noise of surrounding. Technique is reliable in terms of giving quick and repeatable results. This technique is based on the shift in the resonating frequency due to placing sample in front of the probe. For the present studies, we put the sample solution into polypropylene made sample holder (250 µL Holding capacity) then heat it using hotplate for the range 30 °C to 55 °C. To make heating effectively, the isolated box was used for thermal insulation and experimental setup is discussed in supplementary file (See Supplementary File 2). The method of calculating the dielectric constant is also previously reported^23^.

### Mechanical frequency, dipole moment and polarization current density measurements

To further understand phenomenon taking place at molecular level, a theoretical model was developed where it is assumed that the NP protein complex suspended in a buffer medium is governed by three forces (binding, damping and driving forces) and solving for the governing differential equations (See Supplementary File 2) a theoretical expression for mechanical vibration frequency, dipole moment and polarization current density were derived which are dielectric constant dependent (See Supplementary File 2).

1 $$\varepsilon_{r} = 1 + \frac{{NQ^{2} \left( {\omega_{o}^{2} - \omega^{2} } \right)}}{{M\varepsilon_{o} \left[ {\left( {\omega_{o}^{2} - \omega^{2} } \right)^{2} + \left( {\gamma \omega } \right)^{2} } \right]}}$$

2 $$\omega_{o}^{2} = \frac{{\left[ {\frac{{NQ^{2} }}{{M\varepsilon_{o} \left( {\varepsilon_{r} - 1} \right)}} + 2\omega^{2} } \right] \pm \sqrt {\left[ {\frac{{NQ^{2} }}{{M\varepsilon_{o} \left( {\varepsilon_{r} - 1} \right)}} + 2\omega^{2} } \right]^{2} - 4\left( {\frac{{NQ^{2} }}{{M\varepsilon_{o} \left( {\varepsilon_{r} - 1} \right)}} + 2\omega^{2} } \right)\left( {\omega^{4} + \gamma^{2} \omega^{2} + \frac{{NQ^{2} \omega^{2} }}{{M\varepsilon_{o} \left( {\varepsilon_{r} - 1} \right)}}} \right)} }}{2}$$

3 $$\tilde{P}\left( t \right) = \left[ {\frac{{NQ^{2} \left( {\omega_{o}^{2} - \omega^{2} } \right)E_{O} }}{{M\varepsilon_{o} \left[ {\left( {\omega_{o}^{2} - \omega^{2} } \right)^{2} + \left( {\gamma \omega } \right)^{2} } \right]}} + i \frac{{\gamma \omega NQ^{2} E_{O} }}{{M\varepsilon_{o} \left[ {\left( {\omega_{o}^{2} - \omega^{2} } \right)^{2} + \left( {\gamma \omega } \right)^{2} } \right]}}} \right]e^{ - i\omega t}$$

4 $$J_{P} = \frac{{NQ^{2} \left( {\omega_{o}^{2} - \omega^{2} } \right)E_{O} }}{{M\left[ {\left( {\omega_{o}^{2} - \omega^{2} } \right)^{2} + \left( {\gamma \omega } \right)^{2} } \right]}}\left( {\gamma \omega^{2} Cos\left( {\omega t} \right) - \left( {\omega_{o}^{2} - \omega^{2} } \right){{\omega Sin}}\left( {\omega t} \right)} \right)$$

where N, M and Q are number of molecules, mass of molecule and charge on molecule whereas ω, ε_r_ and E_o_ is frequency of drived electric field, dielectric constant, amplitude of electric field. The mechanical frequency (ω_o_), dipole moment (P) and polarization current density (J_P_) of any polar molecule can be calculated by knowing the N, Q, M, ω, ε_r_, E_o_ and ϒ. Where ϒ is known as damping constant and in the present method it is assumed empirical parameter and estimated by the known dipole moment. The dipole moment of insulin and papain are 369 and 150 Debye as reported in literature^42–44^. And for mixing case, ϒ is calculated using the following equation.

5 $$\gamma_{mixed} = \gamma_{1} \frac{{V_{1} }}{{V_{Total} }} + \gamma_{2} \frac{{V_{2} }}{{V_{Total} }}$$

And the factor $\frac{{NQ}^{2}}{M}$ is calculated for the mixing case using the following equation

6 $$\frac{{NQ^{2} }}{M} = \frac{{{\text{N}}_{1} {\text{Q}}_{1}^{2} }}{{{\text{M}}_{1} }} + \frac{{{\text{N}}_{2} {\text{Q}}_{2}^{2} }}{{{\text{M}}_{2} }}$$

The parameters Q and M are perceived through literature ^42–46^. For insulin Q and M are 74.805 × 10^−10^ C and 9.52 × 10^−18^ kg respectively whereas for papain 4.01 × 10^−8^ C and 3.88 × 10^−17^ kg respectively. The value of N is estimated through concentration and volume of the sample and also size and mass of the molecule. ZnO, with its almost 0 net-charge and significantly higher mass compared to insulin and papain, is neglected. The electric field is 4.9 V/m which is calculated using the power delivered by the antenna and the distance between ground plane and tip of the probe. Here the driving frequency is the resonating frequency of probe antenna i.e., 6.41 GHz. Here in equation, the time t is taken 1 microsecond close to protein relaxation time ^47^.

## Supplementary Information

Supplementary Information 1.

Supplementary Information 2.

Supplementary Information 3.
