# Supplementary material for: Role of structural specificity of ZnO particles in preserving functionality of proteins in their corona
Source: Sci Rep. 2021 Aug 5;11:15945. doi: 10.1038/s41598-021-95540-3 (PMC8342705; doi:10.1038/s41598-021-95540-3)
Supplement: Supplementary file 1 — Supplementary Information 1. [file 41598_2021_95540_MOESM1_ESM.docx]

**PROTOCAL OF SAMPLE PREPARATION**

**Precautions**: All vessels forpreparing solutions were autoclaved at 103.42 kilopascal (kPa) at 121 ^o^C for 20 minutes and for cleaning purposes, we have used 75 % of Propan-2-ol (Isopropyl alcohol) in distilled water.

1. **Papain preparations**

Step (A1) Weigh 0.1 g of Papain powder

(A2) Dilute it in 10ml of distilled water and mixed it well.

(A3) To clear the solution, filter it using vacuum pump

(A4) For the lower dilutions with 5 mg/ml (P2) and 2.5 mg/ml (P3), see the Table-1

below:

Table-1

| **Samples** | **Papain Volume** | **Add (for dilution)** | **Concentration** |
| --- | --- | --- | --- |
| **P1** | Solution obtained in (A4) |  | 10mg/ml |
| **P2** | 5ml of P1 solution | 5ml (Distilled water) | 5mg/ml |
| **P3** | 5ml of P2 solution | 5ml (Distilled water) | 2.5mg/ml |

1. **ZnO preparations**

Step (B1) Weigh 3.45 mg of ZnO-Tetrapods (ZnO(T)) and spherical ZnO (ZnO(S))

respectively

(B2) Mix each in 1ml of distilled water separately

(B3) ZnO(T) and ZnO (S)dispersions with a concentration of 3.45 mg/ml are

prepared.

**Step B is summarized in the table below:**

Table-2

| **Samples** | **Take** | **Add** | **Concentration** |
| --- | --- | --- | --- |
| **ZnO(T)** | 3.45 mg | 1ml (Distilled water) | 3.45 mg/ml |
| **ZnO(S)** | 3.45 mg | 1ml (Distilled water) | 3.45 mg/ml |

*ZnO(S) mixes well with water tomake a homogeneous dispersion, but ZnO(T) does not. Therefore, the ZnO(T) dispersion needs to be shaked well before pouring it in the sample holder.

1. **Insulin preparation** (Insulin Humolog (Powder type) purchased from Sigma Aldrich)

Step (C1) Preparation of Buffer: 50.7 ml of distilled water aremixed with 1.3 ml of

HEPES buffer.

(C2) Weigh 6.9 mg of Insulin powder and mix it with 1ml of the buffersolution from

C1.

(C3) Insulin of concentration 6.9 mg/ml is prepared.

**Step C is summarized in the table below:**

Table-3

| **Sample** | **Take** | **Add** | **Concentration** |
| --- | --- | --- | --- |
| **Insulin** | 6.9mg | 1ml of buffer (50.7 ml of Distilled water +1.3 ml of HEPES buffer) | 6.9 mg/ml |

*We are using Insulin as a target protein on which the effect of protease (Papain) was observed at different concentrations, keeping the Insulin concentration equal to the standard dosage of an Insulin-pen (200 U/ml)

1. **Volume consideration for mixing:**Volume ratios of different components are based on the volume of the sample holder, which is 250 μl.

Step (D1) For papain with Insulin (IP1, IP2 and IP3), the volume ratio of Insulin and

Papain Solution is 10:1

(D2) For mixing ZnO with Insulin, we have taken volume ratio of Insulin and ZnO

Sample 2:1

(D3) For Insulin-ZnO-Papain mixture, the volume ratio of Insulin, ZnO and Papain

is 8:4:1

The used amounts of each solution are summarized in the table below:

**Table-4**

| **Mixture Type** | **Ratio (approximate)** | **Volume of insulin sample** | **Volume of Papain sample** | **Volume ZnO sample** |
| --- | --- | --- | --- | --- |
| **IP (for all the three concentrations of papain i.e P1, P2, P3)** | ~10:1 | 230 μl | 20 μl | - |
| **I+ZnO** | ~2:1 | 160 μl |  | 90 μl |
| **I+P+ZnO** | ~8:1:4 | 150 μl | 20 μl | 80 μl |

*ZnO(T) has larger particle size so we had to cut off the micro tip to allow easier sample transfer of the dispersion into the sampleholder.

**Preparations of the samples for Zeta Potential:** For zeta studies, lower dilutions were prepared because high concentrations are not suitable in the Electrophoretcic studies, as they createturbulences. So the 100 µL of prepared solution (ZnO, Papain and Insulin) which were used in dielectric studies, were mixed with 900 µL of distilled water. However, the mixing ratio was same in all experiments.

**Difference between Insulin and Papain:** Results presented in the manuscript under consideration includes two proteins papain and insulin. Both having markedly different properties as shown in Table-5.

Table-5

| **S.No.** | **Property** | **Insulin** | **Papain** |
| --- | --- | --- | --- |
| **1.** | Amino acid residues | 51 ^1^ | 345^2^ |
| **2.** | Molecular Weight | 5808 Da^1^ | 23000 Da^3^ |
| **3.** | Thermal effect | Temperature sensitive^4-7^ | Heat Resistant upto  optimal temperature 60^o^C^8^ |
| **4.** | Function | Hormone^1^ | Protease (A protein which  degrade other proteins)^8^ |

REFERENCE

[1] Mostafa, K., El-Kolaly, M., El-Samaligy, M. & El-Refai, A. Radio biochemical studies on the preparation of insulin radiodiagnostic system. *Arab Journal Of Nuclear Sciences And Applications* **38**, 121–135 (2005).

[2] Lin, M., Yu, T., Wan, J. & Cao, X. Prediction of the Reverse Micellar Extraction of Papain Using Dissipative Particle Dynamics Simulation. *Applied biochemistry and biotechnology* **181**, 1338–1346 (2017).

[3] Seenivasan, R., Roopa, L., Geetha, S., & others. Investigations on purification, characterization and antimicrobial activity of enzyme papain from Carica papaya Linn. *Journal of Pharmacy Research* **3**, 1092–1095 (2010).

[4] Vimalavathini, R. & Gitanjali, B. Effect of temperature on the potency & pharmacological action of insulin. *Indian Journal of Medical Research* **130**, 166–170 (2009).

[5] Chauhan, B. *Principles of biochemistry and biophysics*. (Firewall Media, 2008).

[6] Abramson, H., Gorin, M. & Moyer, L. The Polar Groups of Protein and Amino Acid Surfaces in Liquids. *Chemical Reviews* **24**, 345–366 (1939).

[7] Bekard, I. B. & Dunstan, D. E. Tyrosine autofluorescence as a measure of bovine insulin fibrillation. *Biophysical journal* **97**, 2521–2531 (2009).

[8] https://web.archive.org/web/20140715224627/http://www.biozym.de/datasheets/papain.php
