# Supplementary material for: Role of structural specificity of ZnO particles in preserving functionality of proteins in their corona
Source: Sci Rep. 2021 Aug 5;11:15945. doi: 10.1038/s41598-021-95540-3 (PMC8342705; doi:10.1038/s41598-021-95540-3)
Supplement: Supplementary file 2 — Supplementary Information 2. [file 41598_2021_95540_MOESM2_ESM.docx]

**Experimental Set-up for measuring dielectric properties**

We have performed the experiments with the coaxial fork type probe, the probe is designed at 6.45 GHz and its near field region is less than 10 cm, so it does not sense noise from the surrounding environment. This technique is based on the shift in resonant frequency which is then used to calculate the dielectric constant. Our sample was placed in a sample holder and then heated on hotplate and the antenna response was observed. To make heating effective, an isolation box has been used for thermal insulation. The experimental setup is shown in Figure 1 below. The method of calculating the dielectric constant has also been previously reported^1^. This technique is quick and gives reliable results.


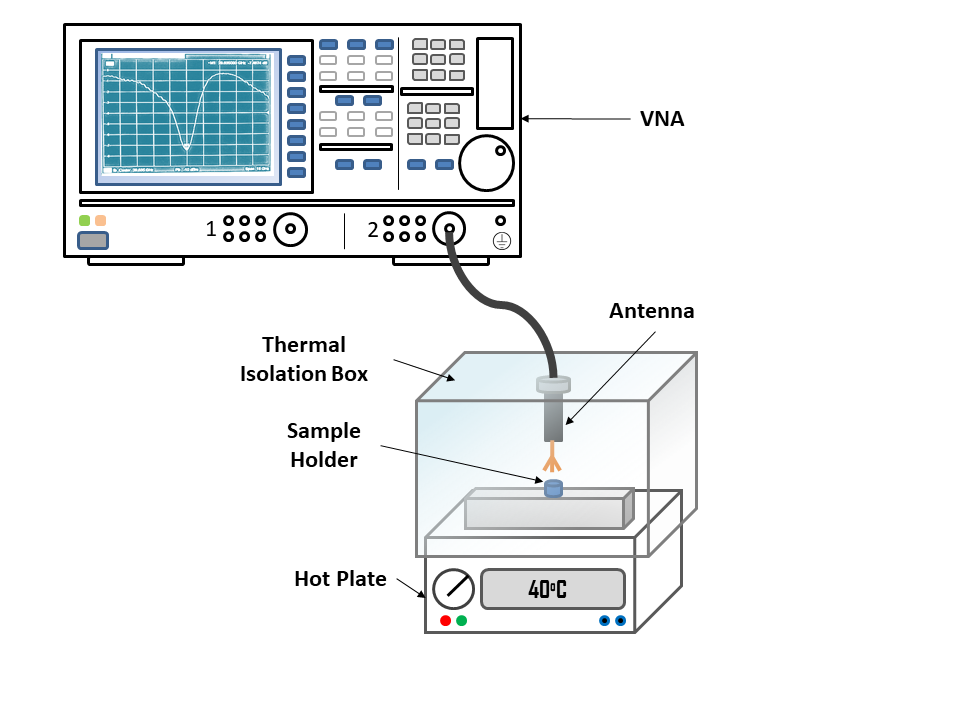


Figure 1 Schematic for Experimental Setup of Resonant Technique

**Method for calculating the dipole moment, polarization current density and mechanical frequency**:

The molecules can be assumed as a set of atoms, arranged in chains and attached by fictitious springs, assuming the force/spring constant K. Binding forces can be represented mathematically by equation 1.

| $F_{Binding}=-Kx=-M\omega_{O}^{2}x$ | (1) |
| --- | --- |

Where $x$ is the displacement from the equilibrium, M is mass of the molecule and ω_o_ is the natural oscillation/frequency of the molecule. In a non-ideal situation, there is a decrease in amplitude of the oscillation, which we often call damping / dragging. In case of bio-molecules, the dragging is encountered by the inertia and viscosity of the solvent. As we know, damping must be opposite in the direction of oscillation and simplest equation which can represent the damping force is following^2^.

| $F_{damping}=-M\gamma\frac{dx}{dt}$ | (2) |
| --- | --- |

Where γ is damping coefficient and t is time. Most of the biomolecules such as proteins and biolipids are polar in nature, so in the presence of the electromagnetic (EM) waves/field, these molecules show many attributes like orientation, electric and ionic polarization. The EM force which drives the polar molecules is called EM driving force. This force depends on the charge of the polar molecules (Q) and the strength of electric field. It can be represented by the following equation^2^.

| $F_{Driving}=QE=QE_{O}Cos(\omega t)$ | (3) |
| --- | --- |

Where E_0_ and ω is the amplitude and driving frequency of the field. By superposition theorem the net force acting on molecule is the sum of the binding force, damping and driving force^2^. For the sake of convenience, we are neglecting the other terms of damping.

| $M\frac{d^{2}x}{{dt}^{2}}=F_{Binding}+F_{Damping}+F_{Driving}$ | (4) |
| --- | --- |
| $M\frac{d^{2}x}{{dt}^{2}}+M\gamma\frac{dx}{dt}+M\omega_{o}^{2}x=QE_{O}Cos\left( \omega t \right)$ | (5) |
| $\frac{d^{2}\tilde{x}}{{dt}^{2}}+\gamma\frac{d\tilde{x}}{dt}+\omega_{o}^{2}\tilde{x}=\frac{QE_{O}e^{-i\omega t}}{M}$ | (6) |

Eq.6 is the second order non homogenous differential equation and one of the solutions of this eq. is (7)^2^

| $\tilde{x}=\tilde{x}_{o}e^{-i\omega t}$ | (7) |
| --- | --- |
| $\tilde{x}_{o}=\frac{\frac{Q}{M}}{\omega_{o}^{2}-\omega^{2}-i\gamma\omega}E_{O}$ | (8) |

The dipole moment of each molecule can be written using the equation (9)^2^.

| $\tilde{p}\left( t \right)=Q\tilde{x}\left( t \right)=\frac{Q^{2}E_{O}e^{-i\omega t}}{M(\omega_{o}^{2}-\omega^{2}-i\gamma\omega)}$ | (9) |
| --- | --- |

The total dipole moment of molecules can be written using the equation (10)^2^.

| $\tilde{P}\left( t \right)=\frac{NQ^{2}E_{O}e^{-i\omega t}}{M(\omega_{o}^{2}-\omega^{2}-i\gamma\omega)}$ | (10) |
| --- | --- |
| $\tilde{P}\left( t \right)=\frac{NQ^{2}\tilde{E}(t)}{M(\omega_{o}^{2}-\omega^{2}-i\gamma\omega)}$ | (11) |
| $\tilde{P}\left( t \right)=\epsilon_{o}\left( 1+\tilde{\epsilon_{r}} \right)\tilde{E}\left( t \right)$ | (12) |

Where N is the total number of molecules. By comparing the equation 11 and 12 , complex permittivity$\left( \tilde{\epsilon_{r}} \right)$can be defined using the equation 13 [2].

| $\tilde{\epsilon_{r}}=\epsilon_{r}+i\epsilon_{i}=1+\frac{NQ^{2}}{M\epsilon_{o}(\omega_{o}^{2}-\omega^{2}-i\gamma\omega)}$ | (13) |
| --- | --- |
| $\epsilon_{r}=1+\frac{NQ^{2}(\omega_{o}^{2}-\omega^{2})}{M\epsilon_{o}[\left( \omega_{o}^{2}-\omega^{2} \right)^{2}+\left( \gamma\omega\right)^{2}]}$ | (14) |

Where $\epsilon_{r}$ and $\epsilon_{i}$ are the real and imaginary part of the permittivity. The equation 14 can be solved further for finding the value of $\omega_{o}^{2}$

| $\omega_{o}^{4}-\left( \frac{NQ^{2}}{M\epsilon_{o}(\epsilon_{r}-1)}+2\omega^{2} \right)\omega_{o}^{2}+\left( \omega^{4}+\gamma^{2}\omega^{2}+\frac{NQ^{2}\omega^{2}}{M\epsilon_{o}(\epsilon_{r}-1)} \right)=0$ | (15) |
| --- | --- |

The equation 15 is quadratic and can be solved using Sridharacharya formula.

| $\omega_{o}^{2}=$ |  |
| --- | --- |
| $\frac{\left( \frac{NQ^{2}}{M\epsilon_{o}(\epsilon_{r}-1)}+2\omega^{2} \right)\pm\sqrt{\begin{aligned} \left( \frac{NQ^{2}}{M\epsilon_{o}\left( \epsilon_{r}-1 \right)}+2\omega^{2} \right)^{2} \\ -4\left( \frac{NQ^{2}}{M\epsilon_{o}(\epsilon_{r}-1)}+2\omega^{2} \right)\left( \omega^{4}+\gamma^{2}\omega^{2}+\frac{NQ^{2}\omega^{2}}{M\epsilon_{o}(\epsilon_{r}-1)} \right) \end{aligned}}}{2}$ | (16) |

The mechanical frequency can be calculated by the equation 16. Further the real part, imaginary part and magnitude of the dipole moment can be defined by the following equations

| $\tilde{P}\left( t \right)=\frac{NQ^{2}\tilde{E}}{M(\omega_{o}^{2}-\omega^{2}-i\gamma\omega)}$ | (17) |
| --- | --- |
| $\tilde{P}\left( t \right)=\left[ \frac{NQ^{2}(\omega_{o}^{2}-\omega^{2})E_{O}}{M\epsilon_{o}[\left( \omega_{o}^{2}-\omega^{2} \right)^{2}+\left( \gamma\omega\right)^{2}]}+i \frac{\gamma\omega NQ^{2}E_{O}}{M\epsilon_{o}[\left( \omega_{o}^{2}-\omega^{2} \right)^{2}+\left( \gamma\omega\right)^{2}]} \right]e^{-i\omega t}$ | (18) |
| $P_{real}=\frac{NQ^{2}(\omega_{o}^{2}-\omega^{2})E_{O}}{M\epsilon_{o}[\left( \omega_{o}^{2}-\omega^{2} \right)^{2}+\left( \gamma\omega\right)^{2}]}\left( \left( \omega_{o}^{2}-\omega^{2} \right)cos(\omega t \right)-\gamma\omega Sin(\omega t))$ | (19) |
| $P_{imag}=\frac{NQ^{2}(\omega_{o}^{2}-\omega^{2})E_{O}}{M\epsilon_{o}[\left( \omega_{o}^{2}-\omega^{2} \right)^{2}+\left( \gamma\omega\right)^{2}]}\left( \left( \omega_{o}^{2}-\omega^{2} \right)Sin(\omega t \right)+\gamma\omega Cos(\omega t))$ | (20) |
| $P_{mag}=\sqrt{P_{real}^{2}+P_{imag}^{2}}$ | (21) |

Using equation 16 and 21, the mechanical frequency and dipole moment of any polar molecule can be calculated by knowing the parameters N, Q, M, ω, εr, E_o_ and $\gamma$. $\gamma$ is known as damping constant and in this method it is estimated by the known dipole moment. The dipole moments of insulin and papain are 369 and 150 Debye as reported in literature^3-5^. By employing method 1, we have already calculated εr. Using the dipole moment and mechanical frequency, the polarization current density also can be calculated by eq. 22.

| $J_{p}=\frac{d\tilde{P}}{dt}$ | (22) |
| --- | --- |
| $J_{p}=\frac{NQ^{2}\left( \omega_{o}^{2}-\omega^{2} \right)E_{O}}{M\left[ \left( \omega_{o}^{2}-\omega^{2} \right)^{2}+\left( \gamma\omega\right)^{2} \right]}\left( \gamma\omega^{2}Cos\left( \omega t \right)-\left( \omega_{o}^{2}-\omega^{2} \right)\omega sin(\omega t) \right)$ | (23) |

For the mixing case, gamma is calculated using the following equation:

| $\gamma_{mixed}=\gamma_{1}\frac{V_{1}}{V_{Total}}+\gamma_{2}\frac{V_{2}}{V_{Total}}$ | (24) |
| --- | --- |

And the factor$\frac{{NQ}^{2}}{M}$ is calculated for the mixing case using the following equation:

| $\frac{{NQ}^{2}}{M}=\frac{N_{1}Q_{1}^{2}}{M_{1}}+\frac{N_{2}Q_{2}^{2}}{M_{2}}$ | (25) |
| --- | --- |

The parameters Q and M are also reported in literature. For insulin Q and M are 74.805X10^-10^ coulomb and 9.52X10^-18^ Kg respectively whereas for papain 4.01X10^-8^ coulomb and 3.887X10^-17^ kg respectively^3-7^. The electric field is 4.9 V/m, which is calculated using the power delivered by the antenna and the distance between ground plane and tip of the probe. Here the metal plane of the hot plate (where the sample holder is placed) is considered as ground plane. The number N is estimated from the volume, concentration of the sample as well as size and mass of the molecule^7,8^. In the antenna experiment, the volume of the sample used in our study is fixed at 250 µl. ZnO with its almost non-existing net charge and significantly higher mass compared to insulin and papain, is neglected. Here in equations, the time t is taken as 1 microsecond, which is close to the proteins relaxation time^9^.

The calculated dipole moment and Polarization current density for the various samples from above model are shown in table.

| Samples | Dipole moment (Debye) | Polarization Current Density (nA/m^2^) |
| --- | --- | --- |
| Insulin | 361 | 7.04 |
| Insulin+Papain | 275.9 | 2.44 |
| Insulin+ZnO(T) | 231 | 4.50 |
| Insulin+ZnO(T)+Papain) | 266.1 | 2.36 |
| Insulin+ZnO(S) | 231 | 4.50 |
| Insulin+ZnO(S)+Papain | 253.9 | 2.25 |
| Papain | 150.5 | 2.93 |
| Papain+ZnO(T) | 96.4 | 1.88 |
| Papain+ZnO(S) | 96.4 | 1.88 |

**Microwave Spectroscopy:**

**Experimental Set-up:** To verify the theoretical values of the mechanical frequencies, a device was printed using a 3D printer (ANYCUBIC Mega i3 FDM), as shown in Fig.2 and Fig.3. PLA filament was used, which is a low cost dielectric material. For making the device conducting, the copper tape was used. For signal efficiency, this adhesive tape was pasted in two parts, where tape of the inner body of device is isolated from the tape of the outer body (see Fig3). For launching the EM waves, SMA female coaxial connecters were fixed on both ends of the device through screws. The panel launcher of the two connectors touches the mid part of the device whereas the ground plane of the connector touches the tape of the outside device. The first and end port of the device is connected to Spectrum Analyzer and PSG vector signal generator relatively through RF coaxial cable. From the signal generator, 10 µW power are delivered to one end of the device. Power is measured using a spectrum analyzer from another port. A copper taped Teflon made cylinder (CTTMC) is placed as a sample holder in the device. The thickness, outer diameter and length of the pipe are 2mm and 4mm and 18.5mm respectively. Another copper tape of thickness 0.14 mm is coated onto the outer surface of the cylinder. These sample holders are disposable and inexpensive. The power of the signal is measured first before loading the sample into the sample holder. In almost all sample holders the power is found to be about 9.1-9.9 µW. This drop in power is due to EM leakage or an air cavity under the copper taping on the device. So, those sample holder are used, in which the power drop is minimum, and the power is observed about 9.8 to 9.9 µW. Samples are heated using the hot plate for two temperatures 45 ^o^C and 50 ^o^C. The mechanical frequencies corresponding to these temperatures are determined for validating the theoretical model.


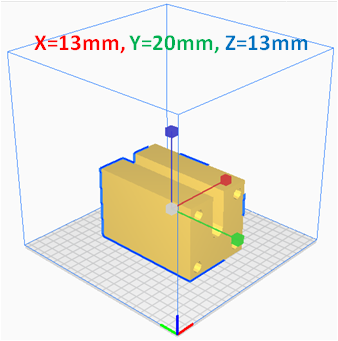


Figure 2 Computer Aided Design (CAD)-view of the Device designed on CST Studio Suite (Academic) 2019. The above image is obtained from the STL file viewed on Ultimaker CURA ver.4.7.1.


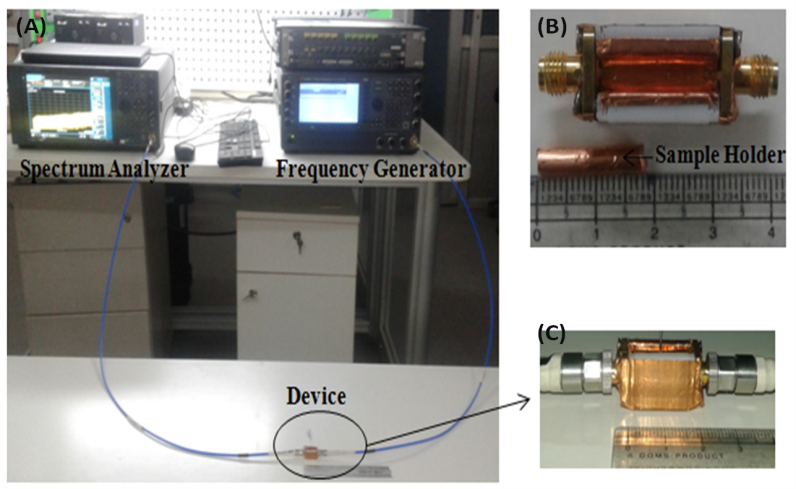


Figure 3(A) Experimental Set-up (B) Top view of the Device and Sample holder (C) Side view of the Device


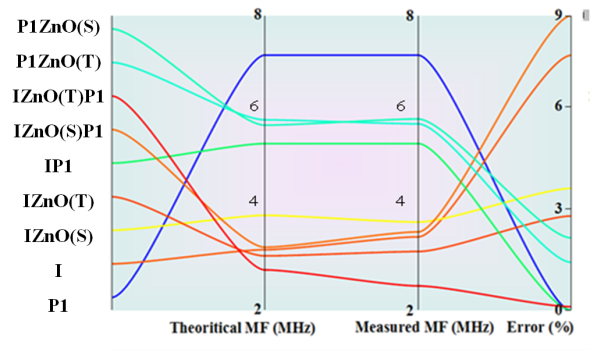

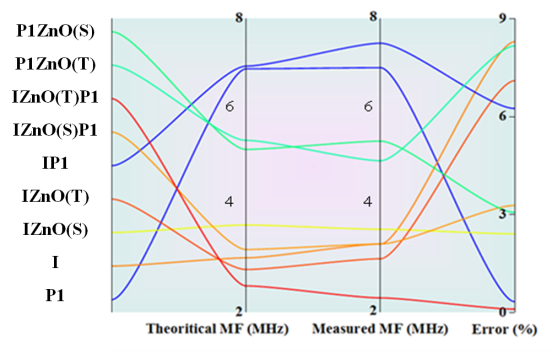


Figure 4 Panel chart of theoretical and measured Mechanical Frequency (on temperature (A) 45 ^o^C and (B) 50 ^o^C respectively)

Error is calculated for comparing the experimental MF with theoretically calculated MF. Experimental results show consistency with the theoretical results. For most of the samples, the error is below 4 %, which suggests the validity of the theoretical results. But for a few samples error is up to 10%. This may be due to the dynamic interaction of papain-insulin–NPS.

REFERENCE

[1] Urvashi et.al. Measurement of Dielectric Properties of Biological Materials Using Co-Axial Fork-Type Probe. *IEEE Sensors Journal* 19, 10482–10489 (2019).

[2] Griffiths, D. J. *Introduction to electrodynamics*. *Chapter9: Electromagnetic Waves* (American Association of Physics Teachers, 2005).

[3] Takashima, S. &Asami, K. Calculation and measurement of the dipole moment of small proteins: use of protein data base. *Biopolymers: Original Research on Biomolecules* 33, 59–68 (1993).

[4] Van Duijnen, P. T., Thole, B. T. & Hol, W. On the role of the active site helix in papain, an ab initio molecular orbital study. *Biophysical chemistry* 9, 273–280 (1979).

[5] Felder, C. E., Prilusky, J., Silman, I. &Sussman, J. L. A server and database for dipole moments of proteins. *Nucleic acids research* 35, W512–W521 (2007).

[6] Waugh, D. F. A fibrous modification of insulin. I. The heat precipitate of insulin. *Journal of the American Chemical Society* 68, 247–250 (1946).

[7] [https://www.bioinformatics.org/sms/prot_mw.html“ for](https://www.bioinformatics.org/sms/prot_mw.html) obtaining the mass of protein by its FASTA sequence”

[8] Erickson, H. P. Size and shape of protein molecules at the nanometer level determined by sedimentation, gel filtration, and electron microscopy. *Biological procedures online***11**, 32–51 (2009).

[9] Pethig, R. R. *Dielectrophoresis: Theory, methodology and biological applications*. (John Wiley & Sons, 2017).
