# Supplementary material for: Role of structural specificity of ZnO particles in preserving functionality of proteins in their corona
Source: Sci Rep. 2021 Aug 5;11:15945. doi: 10.1038/s41598-021-95540-3 (PMC8342705; doi:10.1038/s41598-021-95540-3)
Supplement: Supplementary file 3 — Supplementary Information 3. [file 41598_2021_95540_MOESM3_ESM.docx]

**Data values for Figure 1**

|  | Dielectric Constant | | |
| --- | --- | --- | --- |
| T(^O^C) | P3 | P2 | P1 |
| 30 | 22.03 | 23.71 | 27.17 |
| 35 | 23.71 | 23.71 | 27.17 |
| 40 | 23.71 | 24.57±0.85 | 29.88±0.92 |
| 45 | 25.42 | 26.29±0.87 | 29.88±0.92 |
| 50 | 27.17 | 28.963 | 32.74 |
| 55 | 27.17 | 28.06±0.89 | 30.81 |

|  | Dielectric Constant | | | | | |
| --- | --- | --- | --- | --- | --- | --- |
| T(^O^C) | P3ZnO(T) | P3ZnO(S) | P2ZnO(T) | P2ZnO(S) | P1ZnO(T) | P1ZnO(S) |
| 30 | 32.81±0.39 | 29.77±1.10 | 32.41 | 32.04±1.16 | 34.48±2.06 | 38.33 |
| 35 | 35.37±2.95 | 30.88 | 32.41 | 39.77±1.43 | 36.81±4.39 | 41.21 |
| 40 | 38.87±2.33 | 34.60±3.72 | 36.81±4.39 | 41.21 | 42.71±10.29 | 42.78±1.57 |
| 45 | 41.21 | 39.77±1.43 | 43.92±2.70 | 46.08±1.73 | 47.11±5.89 | 44.35 |
| 50 | 41.21 | 41.21 | 46.62 | 46.08±1.73 | 53.01 | 54.22±6.40 |
| 55 | 41.21 | 39.77±1.43 | 46.62 | 44.35 | 53.01 | 47.81 |

|  | Dielectric Constant | | |
| --- | --- | --- | --- |
| T(^O^C) | I | IZnO(T) | IZnO(S) |
| 30 | 68.60 | 95.57±2.34 | 38.97±14.48 |
| 35 | 92.23 | 128.77±28.83 | 65.46±47.69 |
| 40 | 194.18±7.59 | 178.56±16.72 | 77.57±27.83 |
| 45 | 285.29±10.74 | 266.15±14.48 | 95.57±59.75 |
| 50 | 318.98 | 303.27±56.04 | 150.35±22.63 |
| 55 | 369.44 | 351.60±38.83 | 167.56±25.68 |

|  | Dielectric Constant | | |
| --- | --- | --- | --- |
| T(^O^C) | IP1 | IP2 | IP3 |
| 30 | 35.96±12.95 | 43.15 | 68.60 |
| 35 | 44.76±4.15 | 44.76±4.15 | 161.52±92.91 |
| 40 | 54.60±14.00 | 58.05±5.87 | 205.61±113.37 |
| 45 | 59.97±19.37 | 161.52±92.91 | 311.94±57.50 |
| 50 | 50.13±35.37 | 95.91±3.67 | 321.99±47.44 |
| 55 | 33.47±18.71 | 241.32±54.72 | 413.50±44.05 |

|  | Dielectric Constant | | | | | | |
| --- | --- | --- | --- | --- | --- | --- | --- |
| T(^O^C) | IZnO(T)P1 | | | IZnO(T)P2 | | IZnO(T)P3 | |
| 30 | 48.74±12.11 | | | 48.74±12.11 | | 25.27±3.45 | |
| 35 | 94.30 | | | 133.62±72.77 | | 28.84±3.64 | |
| 40 | 167.56±38.83 | | | 110.05 | | 77.57±16.72 | |
| 45 | 253.01±124.27 | | | 152.60±23.87 | | 85.45±24.60 | |
| 50 | 407.09±166.13 | | | 223.68±17.28 | | 102.17±7.87 | |
| 55 | 475.26±97.96 | | | 377.29205 | | 130.39±20.33 | |
|  | | Dielectric Constant | | | | |  |
| T(^O^C) | | IZnO(S)P1 | IZnO(S)P2 | | IZnO(S)P3 | |  |
| 30 | | 94.30 | 77.57±16.72 | | 36.63 | |  |
| 35 | | 122.51±28.21 | 94.30 | | 48.74±12.11 | |  |
| 40 | | 152.60±23.87 | 130.39±20.33 | | 82.68±46.05 | |  |
| 45 | | 206.39 | 178.56±27.83 | | 150.35±56.04 | |  |
| 50 | | 266.15±59.75 | 223.68±17.28 | | 167.56±38.83 | |  |
| 55 | | 291.84±85.44 | 325.91 | | 178.56±27.83 | |  |

**Data values for Figure 2**

|  | Mechanical Frequency (MHz) | | |
| --- | --- | --- | --- |
| T(^O^C) | I | IZnO(T) | IZnO(S) |
| 30 | 4.591 | 3.790±0.146 | 4.746±0.073 |
| 35 | 4.260 | 3.586±0.350 | 4.304±0.515 |
| 40 | 3.532±0.034 | 3.238±0.127 | 4.011±0.222 |
| 45 | 3.207±0.030 | 2.942±0.168 | 3.790±0.146 |
| 50 | 3.117 | 2.826±0.053 | 3.449±0.339 |
| 55 | 3.005 | 2.723±0.049 | 3.306±0.196 |

|  | Mechanical Frequency (MHz) | | |
| --- | --- | --- | --- |
| T(^O^C) | IP3 | IP2 | IP1 |
| 30 | 4.556 | 5.192 | 5.699±0.552 |
| 35 | 3.915±0.640 | 5.152±0.122 | 5.272±0.125 |
| 40 | 3.661±0.566 | 4.822±0.124 | 5.060±0.337 |
| 45 | 3.128±0.146 | 3.965±0.649 | 4.975±0.423 |
| 50 | 3.097±0.115 | 4.240±0.041 | 5.748±1.282 |
| 55 | 2.904±0.077 | 3.388±0.196 | 6.047±0.984 |

|  | Mechanical Frequency (MHz) | | |
| --- | --- | --- | --- |
| T(^O^C) | IZnO(T)P3 | IZnO(T)P2 | IZnO(T)P1 |
| 30 | 5.343±0.191 | 4.629±0.299 | 4.785±0.309 |
| 35 | 5.160±0.169 | 3.755±0.574 | 4.005 |
| 40 | 4.027±0.223 | 3.726 | 3.495±0.207 |
| 45 | 3.954±0.296 | 3.445±0.136 | 3.264±0.438 |
| 50 | 3.731±0.072 | 3.120±0.060 | 2.854±0.308 |
| 55 | 3.519±0.139 | 2.734 | 2.685±0.140 |

|  | Mechanical Frequency (MHz) | | |
| --- | --- | --- | --- |
| T(^O^C) | IZnO(S)P3 | IZnO(S)P2 | IZnO(S)P1 |
| 30 | 4.838 | 4.102±0.227 | 4.005 |
| 35 | 4.544±0.294 | 3.875 | 3.782±0.223 |
| 40 | 4.177±0.661 | 3.584±0.141 | 3.561±0.141 |
| 45 | 3.463±0.340 | 3.312±0.130 | 3.288 |
| 50 | 3.319±0.196 | 3.120±0.060 | 3.110±0.178 |
| 55 | 3.251±0.128 | 2.836 | 3.057±0.230 |

|  | Mechanical Frequency (MHz) | | |
| --- | --- | --- | --- |
| T(^O^C) | P3 | P2 | P1 |
| 30 | 5.469 | 6.380 | 7.321 |
| 35 | 5.365 | 6.380 | 7.321 |
| 40 | 5.365 | 6.323±0.057 | 7.144±0.057 |
| 45 | 5.269 | 6.212±0.053 | 7.144±0.057 |
| 50 | 5.179 | 6.058 | 6.976 |
| 55 | 5.179 | 6.108±0.050 | 7.086 |

|  | Relative change in Mechanical Frequency | | | | | |
| --- | --- | --- | --- | --- | --- | --- |
| T(^O^C) | P1ZnO(T) | P1ZnO(S) | P2ZnO(T) | P2ZnO(S) | P3ZnO(T) | P3ZnO(S) |
| 30 | 6.166±0.095 | 5.993 | 5.268 | 5.285±0.049 | 4.412±0.013 | 4.525±0.043 |
| 35 | 6.070±0.187 | 5.883 | 5.268 | 4.999±0.046 | 4.332±0.093 | 4.482 |
| 40 | 5.887±0.370 | 5.828±0.054 | 5.110±0.157 | 4.953 | 4.226±0.065 | 4.360±0.121 |
| 45 | 5.699±0.183 | 5.773 | 4.876±0.076 | 4.814±0.046 | 4.161 | 4.200±0.038 |
| 50 | 5.516 | 5.497±0.166 | 4.799 | 4.814±0.046 | 4.161 | 4.161 |
| 55 | 5.516 | 5.663 | 4.799 | 4.860 | 4.161 | 4.200±0.038 |

|  | Relative change in Mechanical Frequency | | |
| --- | --- | --- | --- |
| T(^O^C) | IP3 | IP2 | IP1 |
| 30 | -0.0075 | 0.1309 | 0.2413 |
| 35 | -0.0808 | 0.2093 | 0.2376 |
| 40 | 0.0364 | 0.3652 | 0.4325 |
| 45 | -0.0244 | 0.2364 | 0.5512 |
| 50 | -0.0064 | 0.3599 | 0.8437 |
| 55 | -0.0334 | 0.1276 | 1.0121 |

|  | Relative change in Mechanical Frequency | | |
| --- | --- | --- | --- |
| T(^O^C) | I | IZnO(T) | IZnO(S) |
| 30 | 0 | -0.1745 | 0.0337 |
| 35 | -0.0721 | -0.1582 | 0.0104 |
| 40 | -0.2306 | -0.0831 | 0.1356 |
| 45 | -0.3015 | -0.0826 | 0.1818 |
| 50 | -0.3209 | -0.0933 | 0.1064 |
| 55 | -0.3455 | -0.0936 | 0.1003 |

|  | Relative change in Mechanical Frequency | | |
| --- | --- | --- | --- |
| T(^O^C) | IZnO(T)P3 | IZnO(T)P2 | IZnO(T)P1 |
| 30 | 0.1637 | 0.0082 | 0.0422 |
| 35 | 0.2113 | -0.1184 | -0.0597 |
| 40 | 0.1400 | 0.0550 | -0.0104 |
| 45 | 0.2329 | 0.0743 | 0.0179 |
| 50 | 0.1966 | 0 | -0.0846 |
| 55 | 0.1709 | -0.0900 | -0.1062 |

|  | Relative change in Mechanical Frequency | | |
| --- | --- | --- | --- |
| T(^O^C) | IZnO(S)P3 | IZnO(S)P2 | IZnO(S)P1 |
| 30 | 0.0538 | -0.1065 | -0.1276 |
| 35 | 0.0667 | -0.0903 | -0.1122 |
| 40 | 0.1826 | 0.0148 | 0.0082 |
| 45 | 0.0798 | 0.0327 | 0.0253 |
| 50 | 0.0647 | 0 | -0.0024 |
| 55 | 0.0818 | -0.0560 | 0.0173 |

|  | Relative change in Mechanical Frequency | | |
| --- | --- | --- | --- |
| T(^O^C) | P3 | P2 | P1 |
| 30 | 0 | 0 | 0 |
| 35 | -0.0190 | 0 | 0 |
| 40 | -0.0190 | -0.0089 | -0.0242 |
| 45 | -0.0366 | -0.0263 | -0.0242 |
| 50 | -0.0531 | -0.0506 | -0.0471 |
| 55 | -0.0531 | -0.0426 | -0.0320 |

|  | Relative change in Mechanical Frequency | | |
| --- | --- | --- | --- |
| T(^O^C) | P1 | P1ZnO(T) | P1ZnO(S) |
| 30 | 0 | -0.1583 | -0.1814 |
| 35 | 0 | -0.1709 | -0.1964 |
| 40 | -0.0242 | -0.1759 | -0.1842 |
| 45 | -0.0242 | -0.2021 | -0.1918 |
| 50 | -0.0471 | -0.2092 | -0.2120 |
| 55 | -0.0320 | -0.2215 | -0.2008 |

|  | Relative change in Mechanical Frequency | | |
| --- | --- | --- | --- |
| T(^O^C) | P2 | P2ZnO(T) | P2ZnO(S) |
| 30 | 0 | -0.1743 | -0.1716 |
| 35 | 0 | -0.1743 | -0.2164 |
| 40 | -0.0089 | -0.1917 | -0.2167 |
| 45 | -0.0263 | -0.2151 | -0.2250 |
| 50 | -0.0506 | -0.2077 | -0.2052 |
| 55 | -0.0426 | -0.2143 | -0.2042 |

|  | Relative change in Mechanical Frequency | | |
| --- | --- | --- | --- |
| T(^O^C) | P3 | P3ZnO(T) | P3ZnO(S) |
| 30 | 0 | -0.1932 | -0.1725 |
| 35 | -0.0190 | -0.1924 | -0.1646 |
| 40 | -0.0190 | -0.2122 | -0.1872 |
| 45 | -0.0366 | -0.2102 | -0.2028 |
| 50 | -0.0531 | -0.1964 | -0.1964 |
| 55 | -0.0531 | -0.1964 | -0.1889 |

Relative change for the pure and mixed samples was calculated using the following equations.

Equation: 1

$$Relative change of pure sample \left( atT^{O}C \right)$$

$$=$$

$$\frac{{value of pure sample}_{at T^{O}C}-{value of pure sample}_{at {30}^{O}C}}{{value of pure sample}_{at 30^{O}C}}$$

Equation: 2

$$Relative change of mixed sample \left( atT^{O}C \right)$$

$$=$$

$$\frac{{value of mixed sample}_{at T^{O}C}-{value of pure sample}_{at T^{O}C}}{value of {pure sample}_{at T^{O}C}}$$
